# Accuracy and Prognostic Role of NCCT-ASPECTS Depend on Time from Acute Stroke Symptom-onset for both Human and Machine-learning Based Evaluation

**DOI:** 10.1007/s00062-021-01110-5

**Published:** 2021-10-28

**Authors:** A. Potreck, C. S. Weyland, F. Seker, U. Neuberger, C. Herweh, A. Hoffmann, S. Nagel, M. Bendszus, M. A. Mutke

**Affiliations:** 1 Department of Neuroradiology, Heidelberg University Hospital, Heidelberg, Germany; 2 Institute of Diagnostic and Interventional Neuroradiology, Inselspital Bern University Hospital, Bern, Switzerland; 3 Department of Neurology, Heidelberg University Hospital, Heidelberg, Germany

**Keywords:** Stroke, Brain, Computed tomography, CT angiography, Thrombectomy

## Abstract

**Purpose:**

We hypothesize that the detectability of early ischemic changes on non-contrast computed tomography (NCCT) is limited in hyperacute stroke for both human and machine-learning based evaluation. In short onset-time-to-imaging (OTI), the CT angiography collateral status may identify fast stroke progressors better than early ischemic changes quantified by ASPECTS.

**Methods:**

In this retrospective, monocenter study, CT angiography collaterals (Tan score) and ASPECTS on acute and follow-up NCCT were evaluated by two raters. Additionally, a machine-learning algorithm evaluated the ASPECTS scale on the NCCT (e-ASPECTS). In this study 136 patients from 03/2015 to 12/2019 with occlusion of the main segment of the middle cerebral artery, with a defined symptom-onset-time and successful mechanical thrombectomy (MT) (modified treatment in cerebral infarction score mTICI = 2c or 3) were evaluated.

**Results:**

Agreement between acute and follow-up ASPECTS were found to depend on OTI for both human (Intraclass correlation coefficient, ICC = 0.43 for OTI < 100 min, ICC = 0.57 for OTI 100–200 min, ICC = 0.81 for OTI ≥ 200 min) and machine-learning based ASPECTS evaluation (ICC = 0.24 for OTI < 100 min, ICC = 0.61 for OTI 100–200 min, ICC = 0.63 for OTI ≥ 200 min). The same applied to the interrater reliability. Collaterals were predictors of a favorable clinical outcome especially in hyperacute stroke with OTI < 100 min (collaterals: OR = 5.67 CI = 2.38–17.8, *p* < 0.001; ASPECTS: OR = 1.44, CI = 0.91–2.65, *p* = 0.15) while ASPECTS was in prolonged OTI ≥ 200 min (collaterals OR = 4.21,CI = 1.36–21.9, *p* = 0.03; ASPECTS: OR = 2.85, CI = 1.46–7.46, *p* = 0.01).

**Conclusion:**

The accuracy and reliability of NCCT-ASPECTS are time dependent for both human and machine-learning based evaluation, indicating reduced detectability of fast stroke progressors by NCCT. In hyperacute stroke, collateral status from CT-angiography may help for a better prognosis on clinical outcome and explain the occurrence of futile recanalization.

**Supplementary Information:**

The online version of this article (10.1007/s00062-021-01110-5) contains supplementary material, which is available to authorized users.

## Introduction

Mechanical thrombectomy (MT) is a successful and effective treatment in acute ischemic stroke due to large vessel-occlusion [1, 2]. While treatment decision in extended time from symptom onset to treatment (OTT) relies on defined mismatch criteria [3, 4], patient selection in OTT ≤ 6 h relies typically on the extent of early signs of infarction on non-contrast CT (NCCT) [5]. To quantify these, the Alberta Stroke Program Early Computed Tomography Score (ASPECTS) was established [6].

Still, futile recanalization occurs and, in a small proportion of patients, follow-up imaging after successful recanalization reveals larger infarction than expected from pretreatment imaging. Especially in hyperacute stroke, ischemic changes on acute NCCT can be very subtle [7–9]. So, reduced interrater-reliability was observed for ASPECTS within an onset-time to imaging (OTI) ≤ 100 min [10]. In patients treated with intravenous alteplase, a significant time-to-CT interaction regarding interrater-reliability and prognostic accuracy of ASPECTS has been reported before [11].

We therefore examined the time-dependent reliability and accuracy of ASPECTS by analyzing ASPECTS on acute and follow-up NCCT in patients with complete mechanical recanalization. In this collective, treatment effect is homogeneous and secondary infarct growth is expected to be minimal. We suppose that NCCT-ASPECTS more accurately represents final infarct extent in longer OTI, while it may not identify fast stroke progressors in short OTI. This should apply to both human, observer-dependent ASPECTS reading and observer-independent machine-learning based ASPECTS evaluations. Secondly, collateral supply on CT-angiography has been proven to be a major predictor of final infarct size and clinical outcome before [12–14]. We propose that the collateral status may fill the diagnostic gap from NCCT-ASPECTS in hyperacute stroke and may help to explain futile recanalization.

## Material and Methods

### Patient Characteristics

The study was approved by the local ethics board. In this retrospective, monocenter study, we included 265 consecutive stroke patients between 03/2015 and 12/2019 with occlusion of the main segment of the middle cerebral artery (MCA) with a known, defined symptom onset time who underwent CT imaging prior and 1 day after MT at our institution. Further inclusion criterion was complete or near complete MT (modified thrombolysis in cerebral infarction score = 2c or 3). This was achieved in 141 (53%) patients and 5 patients were further excluded (2 patients due to large space occupying hemorrhagic transformation on follow-up imaging and 3 patients due to severe motion artifacts on NCCT). In total, 136 patients were included in the analysis. Baseline patient characteristics are displayed in supplemental Table 1. Median OTI was 139 min (IQR 81–203 min), median time from imaging to flow restoration was 86 min (IQR 62–119 min). Favorable clinical outcome 3 months after stroke was defined as modified Rankin scale score (mRS) ≤ 2 or mRS = 3 in patients with pre-stroke mRS = 3. Information on mRS was available in 128 (94%) patients.

### CT Imaging

Imaging was carried out on a 64-slice CT scanner (Somatom Definition AS, Siemens Healthineers, Erlangen, Germany). NCCT was acquired at 120 kV with automated adjustment of the tube current. Images were reconstructed by iterative image reconstruction (SAFIRE, Siemens Healthineers) with a kernel of J31 and a slice thickness (ST) of 1 and 4 mm for all patients examined later than 10/2016 (92 of 136 patients, 68%). For the remaining, only images with a ST of 4 mm reconstructed by filtered-back-projection (kernel of H41) were available. Acquisition parameters for single-phase CT-angiography were 120 kV at 20 mA after intravenous administration of a single bolus of 65 mL iodine contrast agent (Xenetix 350, Guerbet, Villepinte, France) followed by a 20 mL saline flush at a flow rate of 4 mL/s.

### ASPECTS Scoring and Evaluation of Collateral Status

ASPECTS were rated on acute and follow-up NCCT by two experienced radiologists (MM and AP, more than 5 years of experience in stroke imaging), blinded to all other imaging and clinical data except for hemispheric stroke lateralization. Additionally, acute ASPECTS was evaluated by machine-learning based e‑ASPECTS Software (e-Stroke Suite Version 9.0, Brainomix Ltd, Oxford, United Kingdom). When available, e‑ASPECTS was evaluated on images with 1 mm ST. In cases where e‑ASPECTS indicated ischemic changes in the unaffected hemisphere, e‑ASPECTS was set to 10. A consensus rating by MM and AP was introduced for the acute and follow-up ASPECTS.

Collateral status was evaluated on CT angiography by a third radiologist (CW, more than 3 years of experience in acute stroke imaging) blinded to all other imaging using the Tan score [15]. In patients who received CT angiography externally before transfer to our hospital, collateral status was evaluated on externally acquired images. Subgroup analysis was carried out with respect to internal or external CT angiography.

### Statistical Analysis

Statistical analysis was performed with R (The R Project for Statistical Computing, V3.1.2). Interrater-reliability and agreement of acute and follow-up ASPECTS were assessed by two-way random effects, absolute agreement intraclass correlation coefficients (ICC(2,1)). Group differences were assessed by Wilcoxon-rank sum test and Kruskal-Wallis test, correlations with Spearman rank-correlation analysis. Univariate and multivariate logistic regressions were carried out for neurological outcome at 3 months. Significance level was set to α = 0.05. Medians are given with their interquartile range (IQR), means with their standard deviation, all confidence intervals (CI) are quoted as 95% CI.

## Results

### Time-dependent Interrater-reliability of ASPECT Soring

We defined three subgroups based on OTI: a) hyperacute stroke with OTI < 100 min (55 patients, 40%); b) imaging between 100 and 200 min (45 patients, 33%) and c) imaging ≥ 200 min after symptom onset (36 patients, 27%).

Interrater reliability was assessed by intraclass correlation coefficient separately between the two raters A and B, as well as separately between the e‑ASPECTS and the consensus rating and, lastly, for all raters together (rater A, B and e‑ASPECTS). Over all raters, lowest interrater reliability was found for the hyperacute setting with ICC = 0.54 (CI 0.39–0.68), compared to ICC = 0.74 (CI 0.61–0.83) for OTI in between 100–199 min and ICC = 0.79 (CI 0.66–0.88) for patients with OTI ≥ 200 min (Table 1).

**Table 1:** Interrater reliability of the acute ASPECTS (Alberta Stroke Program Early Computed Tomography Score) according to the time from symptom onset to imaging. For both human and machine-learning based ASPECTS evaluation, interrater-reliability is found to be lowest in the hyperacute setting with OTI (onset-time to imaging) < 100 min. For the two human raters, it was highest in OTI ≥ 200 min, whereas agreement between the human consensus rating and the e‑ASPECTS was highest for OTI of 100–199 min. (*ICC* intraclass correlation coefficient, *CI* confidence interval)

		Interrater reliability between	
		Rater A and B	Consensus rating and e‑ASPECTS
OTI (min)	*N*	ICC (CI)	ICC (CI)
0–99	55	0.72 (0.57–0.83)	0.57 (0.36–0.72)
100–199	45	0.78 (0.64–0.88)	0.88 (0.79–0.93)
≥ 200	36	0.91 (0.83–0.95)	0.78 (0.59–0.88)
Overall	136	0.79 (0.72–0.85)	0.75 (0.67–0.82)

Noticeably, agreement between the consensus rating and the e‑ASPECTS was highest for OTI of 100–199 min (ICC = 0.88 (0.79–0.93). In the latest time window (OTI > 200 min), agreement between e‑ASPECTS and the consensus rating was higher in patients without prior contrast agent administration (20/36 patients, ICC = 0.82 (0.58–0.92) compared to ICC = 0.74 (0.40–0.89) in patients with prior contrast agent administration and secondary transfer to our hospital). There were no relevant differences regarding prior contrast administration for the two raters or other time windows. Moreover, there was no significant difference in OTI between the two CT reconstruction groups (*p* = 0.06), nor did human ratings (*p* = 0.15) or e‑ASPECTS (*p* = 0.5) differ significantly between these two groups.

### Time-dependent Accuracy of ASPECT Scoring

Median ASPECTS was 9 (7–10) on acute NCCT and 8 (6–9) on follow-up imaging (*p* < 0.001). On a subgroup level, difference between acute and follow-up ASPECTS was evident only in the subgroup with OTI ≤ 100 min (*p* < 0.001), *see* Table 1. Correspondingly, agreement between acute and follow-up ASPECTS improved considerably with longer OTI across all ratings (individual, consensus and e‑ASPECTS, see Table 2). These results did not depend on prior, external contrast-agent administration (supplemental Table 2).

**Table 2:** Agreement between acute and follow-up ASPECTS (Alberta Stroke Program Early Computed Tomography Score) according to the time from symptom onset time to imaging (OTI). Agreement is only moderate in short onset-time to imaging and improves substantially in the later time windows. These findings apply to both human and machine-learning based ASPECTS (e-ASPECTS) evaluation

OTI	Acute ASPECTS	Follow-up ASPECTS	Rater A	Rater B	Consensus-rating	e‑ASPECTS
Min	Median (IQR)	Median (IQR)	ICC (CI)	ICC (CI)	ICC (CI)	ICC (CI)
0–99	9 (8–10)	7(6–9) ^a^	0.47 (0.06–0.71)	0.42 (0.05–0.66)	0.43 (0.02–0.68)	0.24 (0.04–0.49)
100–199	8 (7–10)	8 (6–9)	0.46 (0.15–0.67)	0.48 (0.17–0.69)	0.57 (0.27–0.76)	0.61 (0.34–0.78)
≥ 200	8 (7–10)	8 (7–9)	0.83 (0.67–0.92)	0.77 (0.57–0.88)	0.81 (0.60–0.91)	0.63 (0.22–0.82)
Overall	9 (7–10)	8 (6–9) ^a^	0.51 (0.24–0.69)	0.49 (0.22–0.66)	0.52 (0.22–0.70)	0.42 (0.15–0.61)

^a^ Indicates significant differences

Patients who presented with short OTI had a larger decrease in ASPECTS from acute to follow-up imaging than patients with longer OTI. Importantly, this effect was not confined to the group analysis. Instead, OTI correlated directly with the difference (or shift) between acute and follow-up ASPECTS (ρ = −0.28, CI = −0.43–−0.13), *p* < 0.001 for rater A, ρ = −0.23 (CI = −0.38–−0.07), *p* = 0.008 for rater B, ρ = −0.22 (CI = −0.38–−0.06), *p* = 0.01 for e‑ASPECTS and ρ = −0.33 (CI = −0.47–−0.19), *p* < 0.001 for the consensus rating.

We also noted more decrease (or negative shift) between acute and follow-up ASPECTS as time from imaging to recanalization increased (ρ = 0.20 (0.04–0.37), *p* = 0.02 for the consensus rating and ρ = 0.20 (0.03–0.37), *p* = 0.02 for the e‑ASPECTS rating). No further significant correlations between decrease in ASPECTS and time from imaging to recanalization were found on a subgroup level.

### Collateral Status and OTI

CT angiography was acquired simultaneously with NCCT in 104 patients (76%), whereas 32 patients (24%) underwent external CT angiography and secondary transfer to our hospital. In one patient externally acquired CT angiography was not available for collateral scoring.

**Fig. 1:**
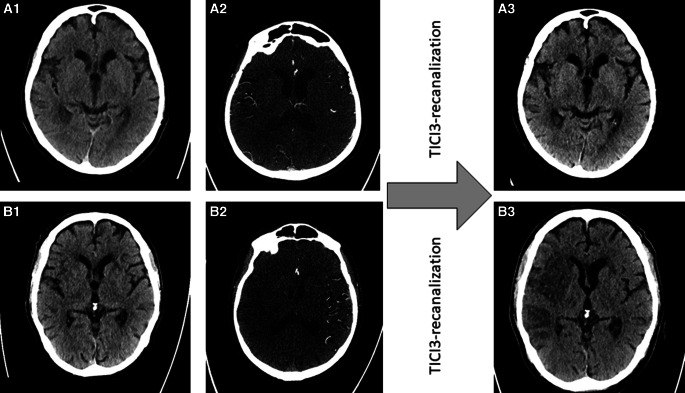
Acute and follow-up imaging of two exemplary patients (A and B) who both underwent acute CT imaging within less than 100 min after symptom onset and who both underwent complete recanalization (mTICI3) of their right-sided middle cerebral artery M1 segment occlusion (patient A within 30 min, patient B within 39 min after image acquisition). On acute NCCT (A1 + B1), early ischemic changes were found for both patients along the right insular ribbon. However, patient A had excellent collateral supply on CT-angiography (A2), while patient B had no collateral filling (B2). On follow-up imaging, large infarction is observed in patient B (B3), while ischemic changes are still confined to the insular ribbon in patient A (A3)

Overall median Tan score was 2 (1–3). Thereby, Tan score was 1 (1–2) in the subgroup with OTI < 100 min, whereas median Tan score was 3 (2–3) in the latest time window with OTI ≥ 200 min (*p* = 0.01). Spearman correlation analysis was significant between Tan scores and ASPECTS (*p* = 0.003 for the consensus rating, *p* = 0.01 for the e‑ASPECTS and *p* < 0.001 for follow-up ASPECTS), see also exemplary patients in Fig. 1. On a subgroup level, correlation was significant only in the early subgroup with OTI < 100 min (*p* = 0.001 for the consensus rating, *p* = 0.003 for the e‑ASPECTS and *p* < 0.001 for the follow-up ASPECTS) and not in the later time windows. This was independent of external or in-house CT angiography acquisition (Table 3 and supplemental table 3).

**Table 3:** Collateral status (Tan score) as a function of OTI (onset-time to image). Significant correlation between Tan score and ASPECTS (Alberta Stroke Program Early Computed Tomography Score) was found especially in the short OTI

OTI		Tan-Score	Correlation between Tan-Score and:					
			Acute ASPECTS (consensus)		e‑ASPECTS		Follow-up ASPECTS	
Min	*N*	Median (IQR)	ρ (CI)	*p*	ρ (CI)	*p*	ρ (CI)	*P*
0–99	55	1 (1–2)	0.42 (0.19–0.64)	0.001	0.39 (0.14–0.63)	0.003	0.57 (0.35–0.79)	< 0.001
100–199	45	2 (1–3)	0.22 (−0.11–0.55)	0.16	0.25 (−0.05–0.56)	0.09	0.25 (−0.09–0.59)	0.09
≥ 200	35	3 (2–3) ^a^	0.14 (−0.23–0.49)	0.44	−0.05 (−0.38–0.28)	0.76	0.20 (−0.16–0.56)	0.25
Overall	135	2 (1–3)	0.25 (0.08–0.42)	0.003	0.21 (0.05–0.38)	0.01	0.39 (0.22–0.55)	< 0.001

^a^ A selection bias towards patients with better collaterals in the later time windows is noted (*p* = 0.01)

### Clinical-outcome Analysis

A favorable clinical outcome 3 months after stroke was observed in 83 of 128 patients (65%). In the univariate analysis, significant predictors of a favorable clinical outcome were ASPECTS (consensus rating: OR: 1.87 (1.44–2.52), *p* < 0.001, e‑ASPECTS: OR 1.77 (1.38–2.34), *p* < 0.001), Tan score (OR: 2.00 (1.37–2.94), *p* < 0.001) and NIHSS at admission (OR: 0.86 (0.80–0.92), *p* < 0.001). In our cohort, OTI was not a significant predictor, nor was time from symptom onset to recanalization (OR 1.00 (1.00–1.01), *p* > 0.5 for both). Also, administration of intravenous thrombolysis (OR 0.84 (0.39–1.76), *p* > 0.5), age (OR 0.97 (0.93–1.00), *p* = 0.1) or sex (OR 1.14 (0.55–2.37), *p* > 0.5) were not significant predictors in our study population. Multivariate analysis including acute ASPECTS, Tan score and NIHSS at admission revealed significant prediction of a favorable clinical outcome for all three (ASPECTS OR: 1.67 (1.26–2.32), *p* < 0.001 or e‑ASPECTS OR: 1.60 (1.23–2.16), *p* = 0.001), Tan score OR: 1.64 (1.05–2.59), *p* = 0.03, NIHSS OR: 0.90 (0.83–0.97), *p* = 0.006.

**Fig. 2:**
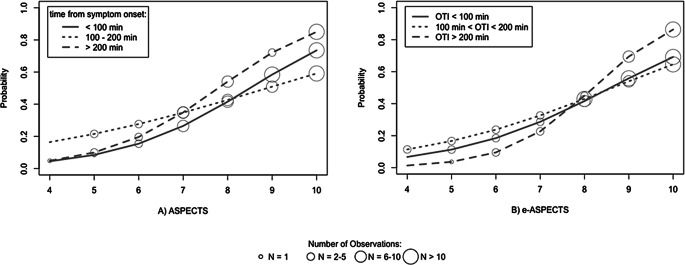
Results of univariate logistic regression analysis: Probability of a favorable clinical outcome 3 months after stroke as a function of **a** observer-dependent ASPECTS and **b** machine-learning based e‑ASPECTS. In patients who presented more than 200 min after symptom onset, ASPECTS was a better predictor of clinical outcome than in earlier time windows for both ASPECTS and e‑ASPECTS

To additionally analyze the time-dependent prognostic roles of NCCT and collaterals, univariate and multivariate logistic regression analysis for a favorable clinical outcome were conducted for the three time-dependent subgroups including ASPECTS and (for multivariate analysis) Tan score. Thereby, pre-interventional ASPECTS was found to be a significant predictor especially in OTI ≥ 200 min, whereas Tan score was also in hyperacute stroke with OTI < 100 min. These findings apply for human observer-dependent ASPECTS and the e‑ASPECTS (see Table 4 and Fig. 2).

**Table 4:** Multivariate logistic regression analysis including either observer-dependent ASPECTS or machine-learning based e‑ASPECTS and collaterals (Tan score) as predictors of a favorable clinical outcome 3 months after stroke. Predictive roles dependent on OTI with collaterals being a significant predictor especially in hyperacute stroke with OTI < 100 min compared to ASPECTS is in the later time windows

OTI	All patients		OTI < 100 min		OTI ≥ 100–199 min		OTI ≥ 200 min	
	OR (CI)	*p*-value	OR (CI)	*p*-value	OR (CI)	*p*-value	OR (CI)	*p*-value
*User-dependent rating:*								
ASPECTS (consensus rating)	1.79 (1.38–2.44)	< 0.001	1.46 (0.91–2.65)	0.15	1.34 (0.86–2.24)	0.2	2.85 (1.46–7.46)	0.01
Collaterals (Tan score)	1.82 (1.22–2.84)	0.004	5.67 (2.38–17.8)	< 0.001	4.04 (1.76–12.3)	0.004	4.21 (1.36–21.9)	0.03
*Machine-learning based ASPECTS evaluation:*								
e‑ASPECTS	1.69 (1.31–2.26)	< 0.001	1.28 (0.72–2.39)	0.4	1.43 (0.97–2.28)	0.09	3.43 (1.66–9.47)	0.004
Collaterals (Tan score)	1.83 (1.22–2.79)	0.004	6.00 (2.52–18.7)	< 0.001	3.85 (1.63–11.8)	0.006	4.03 (1.36–20.6)	0.03

## Discussion

We demonstrate that the reliability and accuracy of NCCT-ASPECTS depend on the time from symptom onset to imaging. Interrater-reliability and agreement between acute and follow-up ASPECTS is found to be reduced in short OTI for both human and machine-learning based ASPECTS evaluation.

A particular strength of this study is the focus on patients who underwent complete mechanical recanalization. Previous works studied the time-dependent accuracy and prognostic value of ASPECTS only in patients treated with intravenous thrombolysis [10, 11, 16]. Compared to these studies, the treatment effect is more homogeneous in our study and the time-dependent accuracy of ASPECTS can be assessed more precisely. We found that patients who underwent imaging within a short OTI exhibited more frequently larger infarcts on follow-up imaging than expected from acute imaging. The most likely explanation is that early ischemic changes on NCCT are only very subtle in patients who present in such short OTI [7–9, 17].

This thesis is further supported by the inclusion of the e‑ASPECTS in our study. e‑ASPECTS did not detect any subtle changes in hyperacute stroke which may be invisible to the human eye. While non-inferiority of e‑ASPECTS was demonstrated before [18], this time-dependency of e‑ASPECTS was not. As an interesting side effect of our analysis, we found that agreement between e‑ASPECTS and the observer-dependent rating was highest in the medium time window with OTI of 100–199 min. One possible explanation could be that e‑ASPECTS is trained most for this time window. Also, we noted a reduced agreement between human consensus rating and e‑ASPECTS in patients with prior external contrast-agent administration. This was more frequent in the latest time window with OTI > 200 min. In those patients, ischemia-related blood-brain barrier disruptions may have led to contrast agent extravasation. Potentially, an enhanced tissue contrast and higher overall density values did then influence the e‑ASPECTS.

Secondly, we investigated whether collateral status from CTA fills the diagnostic gap in hyperacute stroke. Thereby, we found that collateral status correlated with acute and follow-up ASPECTS and clinical outcome after recanalization especially in patients with OTI < 100 min. CTA collaterals allowed for a better prognosis in patients undergoing MT in hyperacute stroke and helped to explain the occurrence of futile mechanical recanalization.

We remark that a third integral part of acute stroke imaging is perfusion imaging. Such imaging is mandatory for patient selection in prolonged time-windows greater than 6 h [3, 4] but current guidelines do not consider it essential for decision-making in shorter time windows [3]; however, the reduced detectability of infarcted tissue by NCCT in the hyperacute setting, as demonstrated in our study, raises the question on whether perfusion imaging may help to identify ischemic tissue especially in the earlier time windows. NCCT-ASPECTS had higher accuracy and reliability in the prolonged time windows for both human and machine-learning based evaluation. Still, in extended time windows, perfusion thresholds have to be met to justify treatment decisions [19]. While our study does not allow for a conclusive analysis on this topic, it strongly motivates for further research on whether NCCT-ASPECTS may be suited as a main selection criterion also in longer OTI. We point out that vice versa, one must not conclude from our study that CT perfusion may be needed in hyperacute stroke for patient selection. Instead, in patients who presented very early after symptom onset and in whom NCCT did not reveal extensive early ischemic changes, a priory probability for a favorable clinical outcome was high as well. For these patients, thrombectomy may be justified per se and regardless of possible infarct underestimation on NCCT. Acquisition of CT angiography is mandatory to diagnose large-vessel occlusion. By assessing the collateral status, we found that it does also allow for a prognosis on the clinical outcome and helps to explain the occurrence of futile recanalization. Conventional CT-perfusion protocols come at the cost of higher radiation dose and the need for more contrast agent. Moreover, CT perfusion may not allow to withhold patients from MT either, due to the risk of infarct overestimation in the hyperacute setting [20–23].

Our study has limitations: First, besides the detectability of early ischemic changes, also infarct growth within the time from imaging until flow restoration leads to a decrease in ASPECTS. Still, this effect was not confined to one of the time windows. Another limitation of our study results from our retrospective, monocentric study design. Compared to a prospective cohort of patients with stroke-like symptoms, the reported accuracies of ASPECTS may be superior. Moreover, we observed a bias towards patients with better collateral status as OTI increased. Noteworthy, this bias occurred although patient selection for MT is not based on collateral status at our institution. Hence, patients with poor collateral supply who presented in a later time window were less likely to be found eligible to undergo MT, possibly due to an already large extent of early ischemic changes on NCCT. This may also explain why correlation between ASPECTS and collateral status was not found to be significant when regarding the extended time window. Further, reconstruction kernel or slice thickness could affect the accuracy of ASPECTS [24, 25] and could not be considered in our retrospective analysis.

## Conclusion

In patients with acute occlusion of the MCA M1 segment who all underwent mechanical recanalization with successful reperfusion, we demonstrate that the accuracy of ASPECTS evaluation is time dependent for both human and observer-independent, machine-learning based evaluation. In ultrashort OTI, collaterals aided to explain the occurrence of futile recanalization. CT-angiography collateral status and NCCT-ASPECTS were complementary surrogate parameters of infarct evolution with different sensitivity regarding time from symptom onset to imaging.

## Supplementary Information

The supplementary information includes patient characteristics, a subgroup analysis of acute and follow-up ASPECTS only for patients who did not receive prior, external sCTA-imaging and a subgroup analysis for patients with in-house CTA-based collateral grading (Tan-score) only.

## Abbreviations

ASPECTS: Alberta Stroke Program Early Computed Tomography Score
CTA: CT angiography
ICC: Intraclass-correlation coefficient
MT: Mechanical thrombectomy
NCCT: Non-contrast computed tomography
NIHSS: National Institutes of Health Stroke Scale
OTI: Onset-time to imaging
OTT: Onset-time to treatment
