# Correction to: Accuracy and Prognostic Role of NCCT-ASPECTS Depend on Time from Acute Stroke Symptom-onset for both Human and Machine-learning Based Evaluation

**DOI:** 10.1007/s00062-022-01158-x

**Published:** 2022-04-07

**Authors:** A. Potreck, C. S. Weyland, F. Seker, U. Neuberger, C. Herweh, A. Hoffmann, S. Nagel, M. Bendszus, M. A. Mutke

**Affiliations:** 1 Department of Neuroradiology, Heidelberg University Hospital, Heidelberg, Germany; 2 Institute of Diagnostic and Interventional Neuroradiology, Inselspital Bern University Hospital, Bern, Switzerland; 3 Department of Neurology, Heidelberg University Hospital, Heidelberg, Germany

**Correction to:**

**Clin Neuroradiol 2021**

10.1007/s00062-021-01110-5

In this article the author name A. Hoffmann was incorrectly written as A. Hoffman. The original article has been corrected.

