# Supplementary material for: Accuracy and Prognostic Role of NCCT-ASPECTS Depend on Time from Acute Stroke Symptom-onset for both Human and Machine-learning Based Evaluation
Source: Clin Neuroradiol. 2021 Oct 28;32(1):133–40. doi: 10.1007/s00062-021-01110-5 (PMC8894298; doi:10.1007/s00062-021-01110-5)
Supplement: Supplementary file 1 — The supplementary information includes patient characteristics, a subgroup analysis of acute and follow-up ASPECTS only for patients who did not receive prior, external sCTA-imaging and a subgroup analysis for patients with in-house CTA-based collateral grading (Tan-score) only. [file 62_2021_1110_MOESM1_ESM.docx]

Supplemental table 1: Patient characteristics. If applicable, median and interquartile range are given.

| Gender (female / male) | 77 / 59 |
| --- | --- |
| intravenous thrombolysis (yes / no) | 83 / 53 |
| age (years) | 78 (68 – 83) |
| CT-Angiography (in-house/external) | 104 / 32 |
| NIHSS at admission | 17 (10 – 21) |
| Time from symptom onset to acute imaging (min) | 139 (81- 203) |
| Recanalization results (mTICI 3 / mTICI 2c) | 104 / 34 |
| Time from imaging to recanalization (min) | 86 (62 – 119) |
| Time from recanalization to follow-up imaging (hh:min) | 21:44 (17:25 – 24:39) |

Supplemental table 2: Subgroup analysis of acute and follow-up ASPECTS, interrater-reliability of acute ASPECTS (both raters and e-ASPECTS) and agreement between acute and follow-up ASPECTS as a function of the time from symptom onset to imaging (OTI) only for patients who did not receive prior, externally sCTA-imaging.

| OTI |  | ASPECTS | | | | | | |
| --- | --- | --- | --- | --- | --- | --- | --- | --- |
|  |  | Consensus-rating | |  | Agreement of acute ASPECTS with follow-up ASPECTS | | | |
|  |  | acute | follow-Up | Interrater-reliability | Rater A | Rater B | Consensus-rating | e-ASPECTS |
| min | N | Median (IQR) | Median (IQR) | ICC (CI) | ICC (CI) | ICC (CI) | ICC (CI) | ICC (CI) |
| 0 – 99 | 55 | 9 (8 -10) | 7(6 – 9) * | 0.55 (0.40 – 0.69) | 0.47 (0.06 – 0.71) | 0.42 (0.05 – 0.66) | 0.43 (0.02 – 0.68) | 0.24 (0.04 – 0.49) |
| ≥100 – 199 | 29 | 8 (7 – 10) | 8 (7 – 10) | 0.69 (0.51 – 0.83) | 0.35 (0.01 – 0.63) | 0.35 (0.01 – 0.62) | 0.47 (0.11 – 0.71) | 0.50 (0.16 – 0.73) |
| ≥ 200 | 20 | 9 (7 -10) | 8 (7 – 9) | 0.85 (0.70– 0.93) | 0.85 (0.64 – 0.94) | 0.80 (0.55 – 0.92) | 0.80 (0.50 – 0.92) | 0.64 (0.14 – 0.86) |
| overall | 104 | 9 (7-10) | 8 (6 – 9) * | 0.64 (0.54 – 0.72) | 0.48 (0.16 -0.67) | 0.44 (0.12 – 0.63) | 0.52 (0.22 – 0.70) | 0.47 (0.14 – 0.67) |

Supplemental table 3: Subgroup analysis regarding only patients with in-house CTA-based collateral grading (Tan-score) according to OTI. Also here, Tan-scores differ significantly between patients with OTI < 100 min and patients with long OTI ≥ 200 min (p = 0.01) with a selection bias towards patients with better collaterals in the later time windows. Significant correlation between TAN-score and ASPECTS (acute and follow-up) were found especially in the short OTI.

| OTI |  | Tan-Score | Correlation between Tan-Score and: | | | | | |
| --- | --- | --- | --- | --- | --- | --- | --- | --- |
|  |  |  | acute ASPECTS (consensus) | | e-ASPECTS | | follow-up ASPECTS | |
| min | N | Median (IQR) | ρ (CI) | p | ρ (CI) | p | ρ (CI) | P |
| 0 – 99 | 55 | 1 (1 – 2) | 0.42 (0.19 – 0.64) | 0.002 | 0.39 (0.14 – 0.64) | 0.003 | 0.57 (0.35 – 0.79) | < 0.001 |
| 100 – 199 | 29 | 2 (1 – 3) | 0.15 (-0.26 – 0.57) | 0.43 | 0.30 (-0.06 – 0.67) | 0.11 | 0.31 (-0.11 – 0.72) | 0.10 |
| >= 200 | 20 | 3 (2 – 3)* | 0.23 (-0.24 – 0.70 | 0.33 | 0.09 (-0.37 – 0.55) | 0.7 | 0.37 (-0.07 – 0.80) | 0.11 |
| overall | 104 | 2 (1 – 3) | 0.28 (0.10 – 0.47) | 0.003 | 0.30 (0.12 – 0.48) | 0.002 | 0.48 (0.30 – 0.65) | < 0.001 |
